# Integrative Volume Status Assessment 

**DOI:** 10.24908/pocus.v7iKidney.15023

**Published:** 2022-02-01

**Authors:** David Kearney, Nathaniel Reisinger, Sadichhya Lohani

**Affiliations:** 1 Renal-Electrolyte Hypertension Division, University of Pennsylvania Philadelphia, PA

**Keywords:** volume overload, hypovolemia, volume assessment, ultrasound, POCUS, physical examination

## Abstract

Volume status assessment is a critical but challenging clinical skill and is especially important for the management of patients in the emergency department, intensive care unit, and dialysis unit where accurate intravascular assessment is necessary to guide appropriate fluid management. Assessment of volume status is subjective and can vary from provider to provider, posing clinical dilemmas. Traditional non-invasive methods of volume assessment include assessment of skin turgor, axillary sweat, peripheral edema, pulmonary crackles, orthostatic vital signs, and jugular venous distension. Invasive assessments of volume status include direct measurement of central venous pressure and pulmonary artery pressures. Each of these has their own limitations, challenges, and pitfalls and were often validated based on small cohorts with questionable comparators. In the past 30 years, the increased availability, progressive miniaturization, and falling price of ultrasound devices has made point of care ultrasound (POCUS) widely available. Emerging evidence base and increased uptake across multiple subspecialities has facilitated the adoption of this technology. POCUS is now widely available, relatively inexpensive, free of ionizing radiation, and can help providers make medical decisions with more precision. POCUS is not intended to replace the physical exam, but rather to complement clinical assessment, guiding providers to give thorough and accurate clinical care to their patients. We should be mindful of the nascent literature supporting the use of POCUS and other limitations as uptake increases among providers and be wary not to use POCUS to substitute clinical judgement, but integrate ultrasonographic findings carefully with history and clinical examination.

## Introduction

Assessment and early optimization of volume status are integral parts of the medical care of critically ill patients especially patients with heart failure, acute kidney injury, and end stage kidney disease (ESKD) on dialysis 1. Decisions on the administration of diuretics, initiation of renal replacement therapy, and ultrafiltration goals rely on proper intravascular assessment. Often, however, in absence of a gold standard, providers’ volume assessments and decisions are not straightforward. For centuries, physicians have relied on the patient’s history and clinical examination findings to come to a differential diagnosis and the most plausible explanation. Yet the accuracy and precision of clinical examination findings are often based on methodologically flawed studies with limitations including small sample size, questionable comparators, and limited generalizability. Thorough clinical examination findings, though important, are often subjective and can lead to bias in decision making process. As the medical field makes progress toward precision medicine, physical examination findings have been supplemented with imaging as well as and invasive modalities such as central venous pressure (CVP) and pulmonary artery (PA) catheter wedge pressure monitoring. More recently, point of care ultrasound (POCUS) examination at bedside has come into widespread practice and has become a fifth pillar of clinical medicine 2. In this review, we discuss integration of conventional methods with newer point of care ultrasound tools for accurate volume assessment during routine care. 

## Conventional Methods of Volume Assessment and Pitfalls

Traditionally, volume status assessment rested on history and physical examination. These methods are readily available, cheap, simple, and non-invasive to the patient and are indispensable in the present time. Even in the era of POCUS, history and physical examination are the clinicians’ first steps in assessing volume status. 

### Mucus Membrane Examination

Mucus membranes of the tongue and oral mucosa are dry in dehydration and intravascular volume depletion. Tongue dryness (P < 0.001) and dryness of the mucous membranes of the mouth (P < 0.001) correlated with severity of dehydration independent of age 3. Dry oral mucosae were associated with hypernatremia (OR = 10.46, 95% CI 6.04 - 18.09) in one case control study 4. Moist mucus membranes and engorged sublingual veins can be present in the context of intravascular volume overload. 

### Capillary Refill Time

Capillary refill time is determined by compressing the distal phalanx of the patient's middle finger positioned level with the heart for 5 seconds and then timing the return of normal color to the finger. The normal capillary refill times are 2 seconds for children and adult men, 3 seconds for adult women, and 4 seconds for the elderly 5, 6. The capillary refill time is also dependent on ambient temperature. Prolonged refill time does not accurately predict hypovolemia (6% sensitivity and 93% specificity) and yields a positive LR of 1.0 7.

### Skin Turgor and Axillary Sweat Examination

Poor skin turgor refers to the slow return of skin to its normal position after being deformed. This can be assessed clinically by pinching the skin between the examiner's thumb and forefinger 8. In a clinical study enrolling elderly patients presenting to emergency departments with vomiting, diarrhea, and/or decreased oral intake presence of dry axilla was only 50% sensitive and 82% specific with a likelihood ratio (LR) of 2.8 for hypovolemia 4. Abnormal skin turgor in the subclavicular area (presence of skin tenting for 3 or more seconds after 3 or more seconds of pinching) was 73% sensitive and 79% specific with a LR of 3.5 for hypovolemia if present 4. The protein elastin, which is responsible for skin recoil, deteriorates with age, limiting the specificity of this test in patients with advanced age 4. 

### Orthostatic Vital Signs

Orthostatic vital signs remain a useful bedside tool to aid in the assessment of volume status. When obtaining postural vital signs, clinicians should wait two minutes before measuring the supine vital signs and one minute after standing before measuring the upright vital signs 5. Orthostatic hypotension is defined as a decrement in systolic blood pressure (SBP) of > 20 mmHg after standing from the supine position 5. In a study of pregnant women presenting to the ED with hyperemesis gravidarum, a 20 mmHg drop in SBP was only 29% sensitive and 81% specific for identifying hypovolemia 9. A drop in SBP by 20 was only 9% sensitive in younger than 65 years and 27% in older than 65 years for detecting moderate blood loss (450-630 ml) 5. The limitation is that orthostatic hypotension has many additional causes beyond volume depletion. This holds particularly true in elderly patients, but also those on vasodilatory agents, those on anti-depressants, and those with a primary autonomic dysfunction like Parkinson’s Disease.

### Jugular Venous Pressure Measurements

Jugular venous pressure (JVP) is measured with patient lying with head of bed at 30-45 degrees with head turned 30-60 degrees away from examiner. The elevation of the neck veins above the sternal angle vertically is measured using tangential light and 5 cm added (right atrium [RA] is 5 cm below the sternal angle) 10, 11. While subject to error, JVP measurements are frequently used for assessment of intravascular volume. In studies comparing JVP measurement to pulmonary artery (PA) catheter measurements, bedside measurements of JVP were within 4 cm of water of the catheter-based measurements 85% on the time 12. Furthermore, a JVP measurement greater than 8 cm of water carries a sensitivity between 47-92%, and a specificity of 93-96% with a positive LR of 9.7 for an elevated central venous pressure (CVP). Conversely, if the measured JVP is less than 5 cm H_2_0, this carries a 90% sensitivity, 89% specificity, and positive likelihood ratio (LR) of 8.4 for a low CVP 12, 13. An additional provocative maneuver, namely testing for hepatojugular reflux, carries a positive LR of 8 for an elevated CVP if positive 14, 15. 

JVP measurement is still fraught with problems. The failure to accurately visualize the JVP by physical examination ranges from 10% to 80% 11, 16. Morbid obesity and a wide neck circumference preclude the internal jugular (IJ) vein from being visualized altogether. An inexperienced operator can frequently misidentify external jugular (EJ) vein for the IJ vein or carotid artery for the IJ vein. Excessive extension or rotation of the chin tenses the sternocleidomastoid muscle leading to compression of the IJ vein and inability to identify this structure. Atrial fibrillation and tricuspid regurgitation can cause the IJ vein to be mistaken for the carotid artery 17. 

### Edema

Peripheral edema is a frequent sign encountered clinically and provides information about interstitial volume. Unfortunately, its presence lacks sensitivity and specificity for the intravascular volume, as numerous disease states can cause edema. These include disease states with increased capillary hydrostatic pressure including volume overload and deep venous thrombosis, but also decreased capillary oncotic pressure as in hypoalbuminemia, increased interstitial hydrostatic pressure as in lymphatic obstruction, and increased capillary permeability as in cellulitis. Therefore, peripheral edema suggests hypervolemia when accompanied by other signs of elevated filling pressures, but in isolation its low sensitivity lacks usefulness 18. The presence of peripheral edema does not necessarily indicate fluid overload and these patients can still be intravascularly depleted or euvolemic 19. In fact, among patients with ESKD, pedal edema correlated with age, body mass index, and left ventricular mass, but did not reflect intravascular volume status 20.

### Lung Examination and Chest Radiograph

Presence of bilateral crackles and rhonchi is suggestive of pulmonary edema 21. Dull percussion is associated with pleural effusion. Pleural effusions indicate increase third space volume. However, for auscultation, the sensitivity is 51% (43-60%), specificity is 79 % (73-84), diagnostic accuracy is 69% (64-74%) for detection of alveolar-pulmonary edema 22. 

Chest x-rays (CXR) are frequently used for assessment of pulmonary vascular status (alveolar edema) characterized by prominent pulmonary veins. The decision in the use of diuretics is commonly made by the presence of alveolar edema on CXR. In a study of 500 chest x-rays in 200 ICUs, CXR findings led to change in therapy in 66% of patients 23. Another less widespread method has been use of upright posteroanterior CXR in describing the relationship of the vascular pedicle width (VPW) and cardiothoracic ratio (CTR) to diagnose cause of pulmonary edema, limited by inability to obtain such view in critically ill ICU patient 24. 

### Biomarkers

B-type natriuretic peptide (BNP) and N-terminal prohormone of BNP (NT-proBNP) has been traditionally used as biomarkers for volume overload or heart failure. At levels < 50 pg/ml, BNP had a negative predictive value of 96% and the diagnostic accuracy for a cutoff of 100 pg/mL was 83.4% 25. Elevated BNP does not always indicate volume overload. Heart failure patients with renal impairment and patients on dialysis have higher concentrations of BNP and NT-proBNP 26. NT-proBNP value cut off 1200 pg/mL for subjects with GFR < 60 ml/min/1.73 m^2^ had sensitivity and specificity to be 89% and 72%, respectively for detecting acute decompensated heart failure 27. On the other hand, BNP testing yielded false negative results in 20% of obese heart failure patients when using the clinical threshold of 100 pg/mL 28. Recent studies have shown serum carbohydrate antigen 125 (CA-125) levels have been associated with state of volume overload and heart failure independently and in combination with NT-proBNP 29, 30, 31.

## Invasive Methods

### Central Venous Pressure (CVP) Measurement

Assessment of CVP is widely available in the intensive care units and is feasible. However, the CVP alone has poor predictive value for fluid responsiveness. A low CVP (mean threshold <8 mm Hg) was associated with fluid responsiveness (positive LR 2.6, 95% CI 1.4-4.6, pooled specificity 76%), but a CVP greater than the threshold made fluid responsiveness less likely (negative LR 0.50, 95% CI 0.39-0.65, pooled sensitivity 62%) 32. A systematic review demonstrated a poor relationship between CVP and blood volume (pooled correlation coefficient 0.16, 95% CI 0.03 - 0.28) and was unable to predict the hemodynamic response to a fluid challenge 33. Several technical aspects of the measurement related to zeroing and leveling of the measuring device can lead to inaccurate measurement of the preload value 34.

### Pulmonary Artery Catheter Pressures

While routine use of pulmonary artery catheter (PAC) has fallen out of favor in the intensive care units (ICU), it is still useful in understanding a patient’s hemodynamics. In one small study, when compared with a physician’s prediction of a variety of hemodynamic variables, PACs were vastly superior. Predictions of RA pressure were accurate approximately 50% of the time 35. However, PAC did not affect overall mortality (OR 1.26; 95% CI, 0.78-2.03; P = 0.35) and hospitalization (HR 1.04, 95% CI 0.86-1.27, P =0.67. Based on the ESCAPE trial, PACs are not routinely indicated to adjust therapy during hospitalization for decompensation of chronic heart failure 36.

### Transpulmonary Thermodilution and Pulse Contour Analysis

Transpulmonary thermodilution (TPTD) and pulse contour analysis are invasive and advanced hemodynamic monitoring techniques used for measurement of cardiac index (CI) and the assessment of cardiac preload by measuring dynamic cardiac preload variable volume variation (SVV) which helps in assessing responsiveness to fluid and pulmonary vascular status. These can be measured accurately only in sinus rhythm and controlled ventilation 37, 38, 39. The TPTD technique provides the variable extravascular lung water index (EVLWI) for the assessment of pulmonary hydration 39, 40. The details of these methods are beyond the scope of this review. These methods are invasive, expensive, and not widely available. 

## Point of Care Ultrasound for Volume Assessment

Point of Care Ultrasound (POCUS) is a bedside ultrasound examination performed by the clinician to answer a focused clinical question. Widespread use of POCUS in the last decade has been shown to improve diagnostic accuracy when used simultaneously with clinical examination 22, 41. Here we describe methods of volume assessment using POCUS as well as their limitations.

### 1. Internal Jugular Vein Assessment

**Figure 1:**
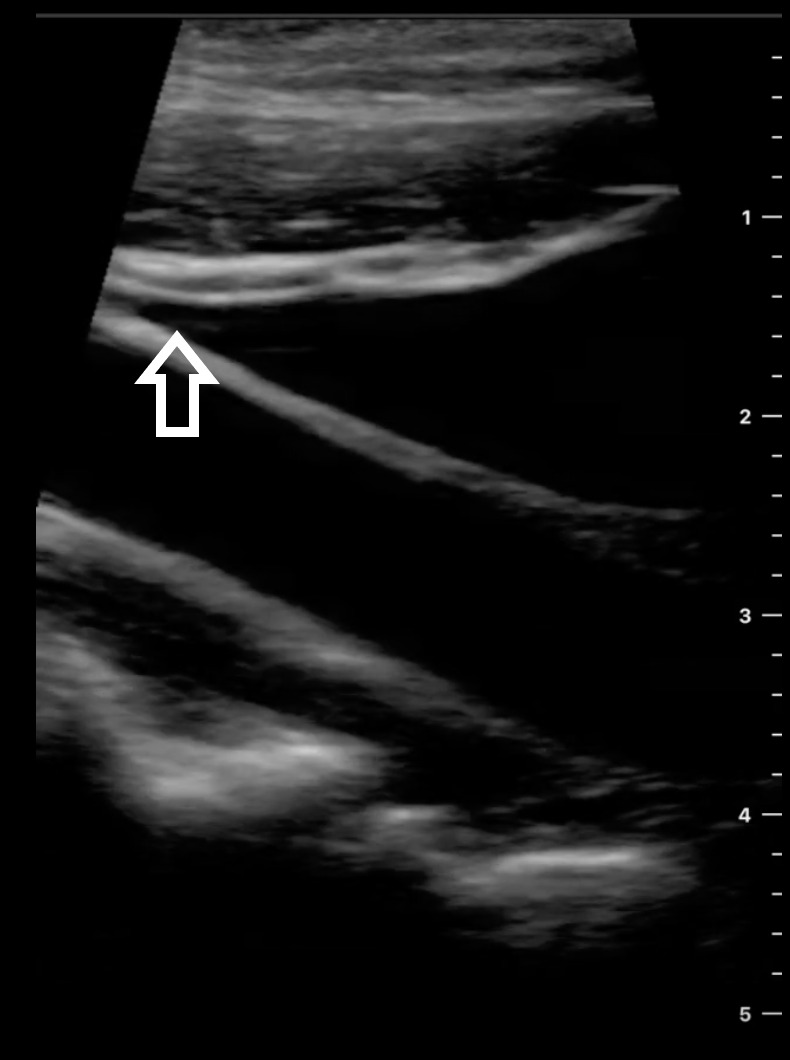
Internal jugular vein showing paint brush sign (arrow).

Ultrasonography of the internal jugular (IJ) vein has been used in the estimation of CVP. The IJ is first identified in transverse section using the linear probe and then the probe is rotated 90 degrees cranially with indicator to the patient’s head. Image of IJ is obtained where IJ narrows into a paintbrush appearance (Figure 1). The height where the IJ tapers correlates with jugular venous distension 42. The IJ diameter is measured using M-mode through several respiratory cycles, and the end expiratory diameter is used as the final measurement. In a small study of non-ventilated patients who were simultaneously undergoing CVP monitoring, a mean IJ vein diameter of 7 mm correlated with a CVP <10 mmHg (95% CI, 5.7-8.3), whereas the IJ vein diameter on 12.5 mm correlated with CVP >10 mmHg (95% CI, 11.2-13.8) 43. Being able to distinguish the IJ vein from the carotid artery is essential to avoid pitfalls. 

### 2. Inferior Vena Cava Assessment

**Figure 2:**
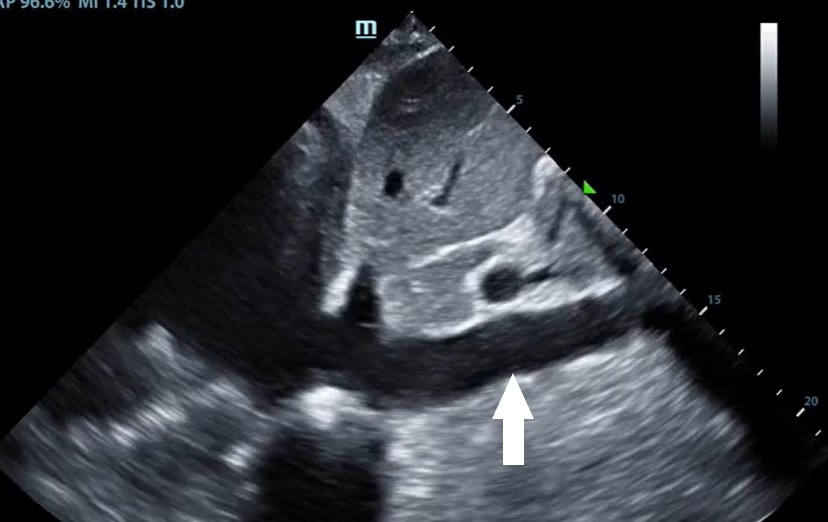
Inferior vena cava. This is subxiphoid view obtained with a linear probe placed below the xiphoid process (white arrow indicating dilated IVC).

Inferior Vena Cava (IVC) measurement is a commonly used ultrasound technique for estimating volume status. To measure IVC, a curvilinear or cardiac probe is placed in the subxiphoid space with transducer flat against the abdomen identifying RA and gradually fanning the probe until the intrahepatic IVC can be identified. The probe is then rotated 90 degrees to obtain the IVC in long axis view. IVC diameter is measured 2 cm inferior to the cavoatrial junction or about 1 cm inferior to the branching of the hepatic veins (Figure 2) 42, 44. M-mode can be used to track IVC collapse during inspiration in spontaneously breathing patients 42.

Measurements used are IVC diametercavalindex whichIVC) minus inspiratory IVC diameter (IVCIVCcavalcavalcavaland the minimum diameter, normalized by the mean of the two values was associated with an increase of cardiac output after fluid resuscitation 45.

The pooled sensitivity and specificity for an IVC ultrasound as a predictor of fluid responsiveness were 0.63 (95% CI 0.56-0.69) and 0.73 (95% CI 0.67-0.78) respectively with a pooled area under the receiver operating characteristic curve of 0.79 (standard error 0.05) 46.

There are many pitfalls when examining the IVC. The abdominal aorta is frequently mistaken for the IVC by inexperienced examiners. IVC and aorta are best distinguished by noting the hepatic veins joining the IVC and the intrahepatic course of the IVC 42. Severe tricuspid regurgitation, tricuspid stenosis, massive pulmonary embolism, cardiac tamponade, restrictive and constrictive cardiomyopathy, pulmonary hypertension, impaired IVC drainage to heart, IVC thrombosis, congestive heart failure, impaired right atrial filling and positive pressure ventilation are commonly encountered scenarios which can lead to a low caval index 47, 48. Changes in intrabdominal pressure, changes in tidal volume on mechanical ventilation, thrombosis of IVC, and presence of an IVC filter are some factors that can affect the IVC diameter and changes seen with inspiration 19. Sometimes patients are unable to tolerate a probe over the subxiphoid space due to abdominal pain or after major abdominal surgeries. The most common error in IVC assessment is failure to identify a slit-like IVC when it is underfilled 42.

### 3. Lung Ultrasound

**Figure 3:**
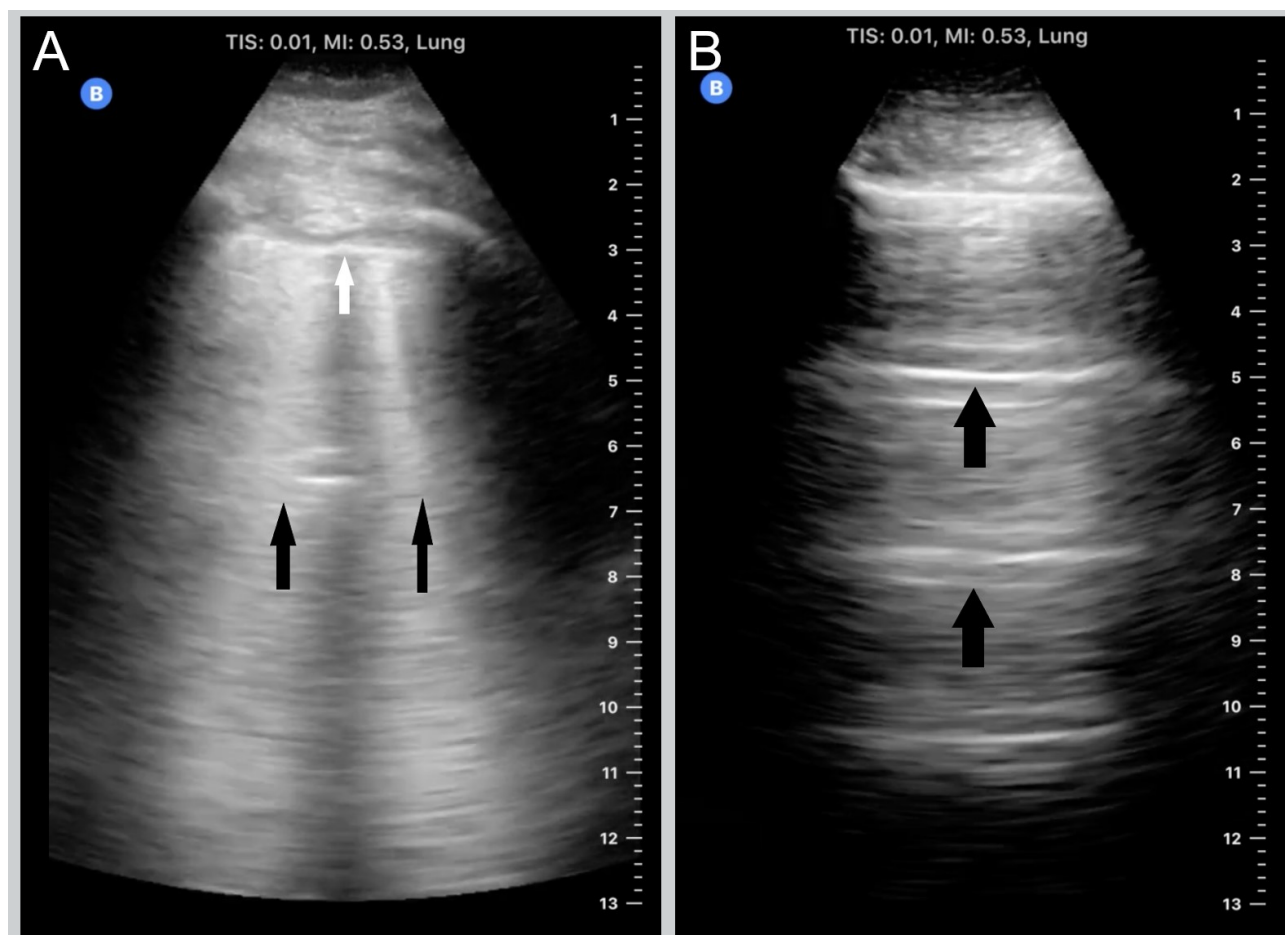
Lung US. A) demonstrating B lines (black arrows) and pleural line (white arrow). B) demonstrating A lines (black arrows)

The volume assessment using lung ultrasonography (LUS) involves examining the patient for B-lines. To evaluate for B-lines, linear probe is used in a vertical configuration placed over the intercostal spaces in the mid-clavicular and mid-axillary lines. B-lines are hyperechoic vertical lines extending from the pleura down to the bottom of the US image (Figure 3). Two or fewer B-lines in each section is considered normal. Alveolar-interstitial syndrome was defined as the presence of more than 3 B-lines or “white lung” appearance for each examined area. Lung pulse was defined as the absence of lung sliding at the pleural line 49.

A meta-analysis showed that LUS is 88% sensitive and 90% specific for acutely decompensated heart failure and was more sensitive at detecting pulmonary edema than CXR 50. LUS B-lines have a high interobserver reliability (concordance index = 0.96) in patients on hemodialysis 51. In dialysis patients, B-lines might be an early marker of extravascular lung water and are likely present before clinical symptoms of dyspnea appear 52. Thirty-four of 40 patients had statistically significant reductions in the number of B-lines from pre-dialysis to the midpoint scan and from pre-dialysis to post-dialysis with a P value < 0.001 52. Patients with an ejection fraction < 50% had pre-dialysis B lines of 45 ± 37; those with ejection fractions > 50% had pre-dialysis B lines of 18 ± 17 52.In the Lung Water by Ultra-Sound Guided Treatment to Prevent Death and Cardiovascular Complications in High Risk ESRD Patients with Cardiomyopathy (LUST) trial, the median number of LUS B-lines before a regular hemodialysis session was 9, and the inter quartile range spanned from 5 to 19 lines 53, estimating to median accumulation of water in the lungs of about 1.2 L in a range comprised between 0.5 and about 2.2 L 54. The number of LUS B-lines decreased in 79% of patients after dialysis and did not change in 21% of patients (i.e., remained exactly the same or changed by two US-B lines at most) 53.

While heart failure is one of many causes of pleural effusions, pleural ultrasound remains a useful tool in assessing volume status. Pleural effusions are easily visualized with a bedside ultrasound by placing a curvilinear probe in the midaxillary line. Pleural effusion can also be detected in cardiac views. Characteristically, left-sided pleural effusion appears posterior and lateral to the descending aorta in parasternal long axis view (PLAX) and apical 4-chamber views (A4C) 55, 56, 57. In subcostal views, a right pleural effusion can also be visualized besides the right cardiac chambers, extending over the bare area of the liver 57.

A meta-analysis determined the sensitivity and specificity of ultrasound for detection pleural effusions as 93% (95% CI, 89% - 96%) and 96% (95% CI, 95% - 98%), respectively 58. The sensitivity approaches 100% with pleural effusions >100 mL in volume. 

However, isolated LUS will not be adequate to make diagnosis of pulmonary edema as the LUS findings can been seen in acute respiratory distress syndrome (ARDS), chronic interstitial lung disease, pneumonia, or other inflammatory conditions which may be difficult to distinguish from that of cardiogenic pulmonary edema 19. The presence of heterogenous distribution of B-lines with spared areas of normal lung in the anterior lung field and homogenous compact B-lines giving appearance of white lung and consolidations with air bronchograms in the posterior lung, reduced lung sliding, and irregular or thickened pleural lines is suggestive of ARDS/pneumonia while homogenous B-lines in anterior and posterior lung fields with absence of pleural irregularity/thickening are consistent with cardiogenic pulmonary edema 49, 59. Patient positioning may also limit from obtaining adequate lung scan zones for comprehensive evaluation 19.

### 4. Focused Cardiac Ultrasound

Focused cardiac ultrasound (FoCUS) can provide a vast amount of data which can be used to interpret volume status. FoCUS allows for rapid evaluation of the “five Es:” pericardial **e**ffusion, qualitative assessment of left ventricular **e**jection, right and left ventricular **e**quality (e.g., right ventricle enlarges due to pulmonary hypertension or embolism), **e**xit (aortic root diameter), and **e**ntrance (IVC) 60. 

**Figure 4:**
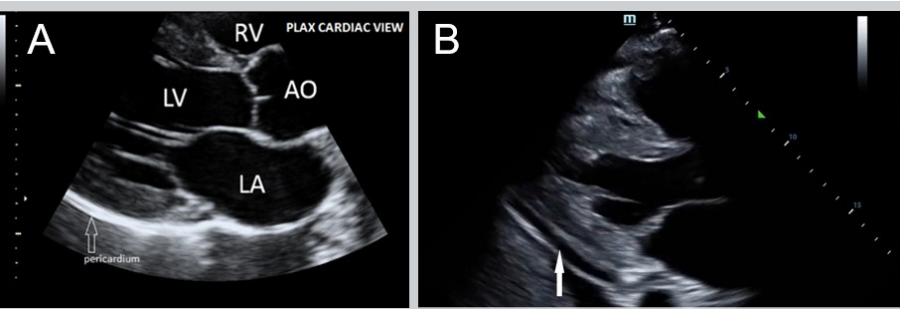
A) Focused cardiac ultrasound (FoCUS). Parasternal long axis view (PLAX) showing different chambers and aorta. B) Focused Cardiac Ultrasound. PLAX view showing pericardial effusion (white arrow).

FoCUS involves PLAX, a parasternal short axis (PSSA), an A4C, and a subcostal 4 chamber or subxiphoid view 60.PLAX view is obtained by placing probe along the left sternal border at 2^nd^ to 4^th^ intercostal space, perpendicular to the patient’s chest with the probe index marker angled toward the patient’s right shoulder and PSSA view by rotating the probe 90 degrees with index toward the left shoulder 44, 48, 61. PLAX view allows visualization of left atrium (LA), mitral valve (MV), left ventricle (LV), aortic valve, right ventricle (RV), pericardium, and descending aortic root (Figure 4) 44. A4C view is obtained with the transducer near the apex of the heart at the point of maximal impulse and directing the index marker toward the patient’s left shoulder to obtain a simultaneous view of the LV, the RV, the LA, the RA, the MV, and the tricuspid valve 48. Subxiphoid or subcostal 4-chamber view is obtained by placing the probe in subxiphoid area as described above during IVC assessment 48. 

Pericardial effusion is seen in several different focused cardiac views, but subxiphoid view provides the clearest and most reliable image 48, 60. In the PLAX and PSSA view, significant effusions are visualized posterior to the LV and anterior to descending aorta and in the A4C view, small effusions are visible lateral to the LV free wall, while moderate to large effusions may be seen around the apex of the heart 60. Small effusions are smaller than 1 cm, moderate effusions are 1 to 2 cm, and large effusions are >2 cm 60, 62. Findings suggestive of cardiac tamponade include, in sequence, collapse of the RA followed by collapse of the RV, and finally LV collapse 63, accompanied by plethoric non collapsible IVC (fifth “e” described above) 60, 64. POCUS was able to demonstrate pericardial effusion with a sensitivity of 96% and a specificity of 98% in emergency room 65. A common pitfall of assessment of pericardial effusion in US is mistaking epicardial or pericardial fat for pericardial effusion 66. 

LV ejection fraction (LVEF) is estimated by qualitative assessment for evaluation for cause for hypotension, chest pain, and dyspnea 60. LVEF can be estimated using PLAX view by the MV E-point septal separation method (EPSS) with visual assessment or measurement of smallest distance between anterior MV and the interventricular septum. Movement to within 1 cm of the septum suggests that the LVEF is likely preserved 44, 48, 60, 61, 67, 68. In PSSA view, concentric LV squeeze can be observed by estimating the degree of internal chamber collapse in systole versus diastole 60. A severely depressed ejection fraction, when combined with a plethoric IVC (and/or B-lines), indicates systolic heart failure 60. Pitfalls include presence of septal hypertrophy, mitral stenosis, atrial fibrillation, and mismeasurement 60. 

Quantitative assessments can also be done in FoCUS for volume assessment. LV internal diameter at the end of diastole (LVIDD) is measured in the PLAX view with the diameter 1 cm distal to the MV annulus in M-mode (reference ranges for LVIDD 3.9 to 5.3 cm in women and 4.2 to 5.9 cm in men); smaller diameters are suggestive of hypovolemia 69. LV end-diastolic area <10 cm^2^ is suggestive of hypovolemia and > 20 cm^2^ indicates volume overload. It is important to note that LV hypertrophy can cause a low LVIDD and therefore lead to erroneous assumptions of volume status 70. Doppler transmitral flow can also be obtained with POCUS and is related to LV filling patterns 71. Interpretation of transmitral flow patterns are confounded by mitral and aortic valve disease and are outside the scope of this article.

The relative size of RV and LV (equality) can be assessed best in PSSA view at the level of papillary muscle where the septal flattening, resulting in the characteristic “D-shaped” LV suggestive of elevated RV pressure can be visualized in RV pressure overload. The normal RV:LV diameter ratio is <0.6:1. Acute RV dilation with or without RV hypokinesis can be seen in pulmonary embolism and also helps predict severity 60. The tricuspid annular plane systolic excursion (TAPSE) is used for estimating the right ventricular systolic function and is best demonstrated in A4C view 60, 72. The TAPSE is obtained by putting the M-mode cursor along the lateral part of the tricuspid valve ring. A TAPSE of 18 mm or greater is typically considered normal 73. The area under the curve of the TAPSE for the detection of CVP greater than 8 mmHg was 0.860 (95% CI 0.730–0.991, P = .001). TAPSE has the potential to predict the CVP in low LVEF patients and provides a noninvasive way to assess the right atrial pressure 72. One of the common pitfalls of RV assessment is overestimation of RV:LV ratio due to the ultrasound plane cutting through the RV in an oblique plane that makes the RV look relatively larger than the LV 60. 

The fourth “E” mentioned above is used for the assessment of the aortic root for thoracic aortic aneurysm and thoracic aortic dissection (exit from the heart) 60. Their description is beyond the scope of this review as this is not pertinent to nephrologist for evaluation of volume status. 

### 5. Doppler Ultrasonography

Doppler ultrasonography evaluation of blood flow pattern in hepatic, portal, and intrarenal veins provides an additional tool for assessing volume status and venous congestion in critically ill patients 45. The probe is placed over the liver in the subcostal position to visualize the middle hepatic vein 74. Pulsed-wave Doppler is used 2-4 cm from where the hepatic vein drains into the IVC. The waveform of the hepatic vein is reversed with higher velocities seen in diastole in states of volume overload. In severe volume overload, retrograde flow is seen in systole 19.

Moving towards the portal vein, the transducer is placed in the right mid-axillary line 74. Flow through the portal vein is normally monophasic, but in the presence of hypervolemia, pulsatility will be present. This can be quantified using the pulsatility index where a pulsatility index greater than 50% indicates severe volume overload 19, 75. Pulsatility can be seen in cirrhosis in absence of elevated CVP due to arterio-portal shunting 76. It can also be observed in healthy, thin adults 77. 

The intrarenal veins, though small and difficult to visualize, can aid in assessing volume status. The curvilinear transducer is placed on the posterior axillary line. A normal Doppler waveform is continuous. With increasing venous congestion, there is a decrease in the systolic component of the wave with progression to biphasic (systolic/diastolic phases), and with severe renal congestion, there is complete absence of systolic flow showing only diastolic phase 78. 

Using the above Doppler methods, venous excess ultrasound score (VEXUS) scoring system was developed to identify venous congestion that leads to distension of IVC hepatic vein, portal vein, and renal vein abnormalities in acute kidney injury (AKI) due to cardiorenal syndrome 79, 80, 81. 

Changes in the common carotid artery (CCA) blood flow using Doppler ultrasonography with response to passive leg raise (PLR) has been shown to be an effective tool in predicting fluid responsiveness in hemodynamically unstable patients 82, 83. Passive leg raise (PLR) is a measure of endogenous volume challenge where the fluid shift from lower extremity to intrathoracic compartment increases cardiac preload by redistributing approximately 300–400 mL of blood from the lower extremities to the heart and subsequent improvement in blood pressure 39, 84. An increase of carotid blood flow by 20% after PLR is indicative of fluid responsiveness with a sensitivity of 94% and specificity of 86% 82. These patients with positive PLR respond to intravenous fluid 84. 

We have summarized the sensitivity, specificity, negative predictive values, and positive predictive values of commonly used physical examination and POCUS findings in Table 1. 

**Table 1:** Sensitivity/specificity in assessment of intravascular volume (volume overload)

**Method of volume assessment**	**Sensitivity**	**Specificity**	**Positive predictive value**	**Negative predictive value**	**Diagnostic accuracy**
Tachycardia^4^	17%	94%			
Orthostatic hypotension 9	29%	81%			
Dry oral mucosa 4	49%	87.8%			
Jugular Venous Pressure >8 cm^12^, ^13^	47-92%	93-96%			
Axillary sweat 4	50%	82%		84%	
Abnormal skin turgor (sub clavicular) 4	73.3%	79%			
Abnormal skin turgor (forearm) 4	68.3%	67.8%			
Auscultation for crackles 22	51% (43-60%)	79 % (73–84%)			
Crackles for severe congestion 53	9%	98%	100%	78%	
Crackles and edema for severe congestion 53	13%	97%	90%	33%	
Chest X-ray 85	46%	80%	Not available	Not available	58%
CVP 32	76%	62%			
IVC assessment (Respiratory variation)^46^	63%	73%	Not available	Not available	
Lung US 50	88%	90%	Not available	Not available	
Carotid blood flow with Passive leg raise 39, 82	94%	86%			

## Integration of Clinical Assessment and POCUS

Conventional physical examination has limited diagnostic utility with poor specificity and sensitivity to predict the intravascular volume status as well as pulmonary congestion and pulmonary volume status in hemodynamically unstable patients 39. 

Several studies have compared the physical examination findings to POCUS findings to understand and improve the diagnostic accuracy of these findings. In a study by Saha et al. in the outpatient cardiology clinic, 51% patients were noted to be euvolemic by IVC assessment and clinical examination while the discordance between IVC assessment by POCUS and JVP assessment by physical examination was 32%, with POCUS proving more accurate 86. In another study in the outpatient hemodialysis unit, 39% of patients who presented at or above dry weight were hypovolemic while 10% of the patients who left HD at or below goal weight were still hypervolemic by IVC assessment 87. 

Similarly, the LUST study demonstrated that peripheral edema was absent in as many as 87% and 80% of assessments where lung US indicated moderate and severe lung congestion, respectively 53. Peripheral edema had virtually no discriminatory power (moderate congestion: AUC 0.54; 95% CI, 0.50 - 0.58; P = 0.05 and severe congestion: AUC 0.56; 95% CI, 0.50 - 0.62; P = .03) 53. The study also found a poor agreement between US B-lines and pulmonary crackles. Crackles had a limited discriminatory power for the diagnosis of mild, moderate, or severe lung congestion as assessed by US (mild lung congestion: AUC = 0.61; 95% CI, 0.57 to 0.64; P = 0.001; moderate congestion: AUC = 0.65; 95% CI, 0.61 to 0.70; P = 0.001; severe congestion: AUC 0.68; 95% CI, 0.62 - 0.74; P = 0.001) 53. The combination of crackles and peripheral edema showed a satisfactory to low positive predictive value (ranging from 79% for mild congestion to 33% for severe congestion)and a high to moderate negative predictive power (ranging from 90% for severe congestion to 48% for mild congestion) 53.

With the limitations of the conventional methods compared to US techniques, US is used more frequently with better predictive capability and objective assessment of volume. However, misdiagnosis based on POCUS findings especially due to limitations of US and lack of experience can also lead to fatal errors in management 57. Hence, it is imperative that we do not rely on one single tool, but rather integrate both pertinent physical examination and POCUS findings for better probability of coming to the right diagnosis. Several studies have suggested such integrative methods. 

US IVC assessment of volume can be limited when used alone as described earlier. However, focusing management based solely on lung US can also sometimes result in intravascular volume depletion. Hence integrating both IVC and lung US has been proposed by Thomas et al. in more accurate estimation of dry weight in hemodialysis patients as IVC and lung US measure intravascular compartment and extravascular lung water respectively 88. Lung and IVC US done immediately before and 30 minutes after the 4th session of hemodialysis based on clinically defined dry weight showed that the number of B-lines reduced from 12.7 ± 9.7 to 4.8 ± 6.6 (P < 0.001) and IVC collapsibility index increased from 0.23 ±0.09 to 0.53± 0.16 (P < 0.001). The coefficient of correlation between reduction in the number of B-lines after HD and change in the IVC collapsibility index was 0.33 (P = 0.004). Basal crackles were present in only 19% of patients with B-lines ≥4 but none of the patients with B-lines ≤4 had crackles 88. 

Tri-POCUS approach in combining lung US, focused cardiac ultrasound, and venous Doppler ultrasound for accurate volume assessment was proposed by Koratala et al. to overcome the limitation of individual methods 19. Integrated cardiopulmonary ultrasound with assessment of cardiac contractility, IVC assessment, and lung US shortened the time to definitive diagnosis of pulmonary edema (17 ± 6 versus 104 ± 34 minutes, P ≤ 0.001) in a study of 128 patients 89, 83. 

Another tool that uses integration of various US methods is the VEXUS scoring system described above which integrates the IVC, hepatic vein waveform and portal vein pulsatility, renal vein Doppler for better assessment of venous congestion and fluid overload. The integration of these 4 parameters would negate the pitfalls of the individual method. In a study of 30 patients, resolution of AKI injury showed significant correlation with improvement in the VEXUS grade (P = 0.003), with significant association between changes in the VEXUS grade and fluid balance (P = 0.006) and a significant difference in fluid balance between the VEXUS score improving group (0.20 ± 1.24 L /day) compared to the no change group (1.67 ± 1.03 L/day) and the worsening group (1.00 ± 0.00 L/day, P = 0.03) 79. 

**Figure 5:**
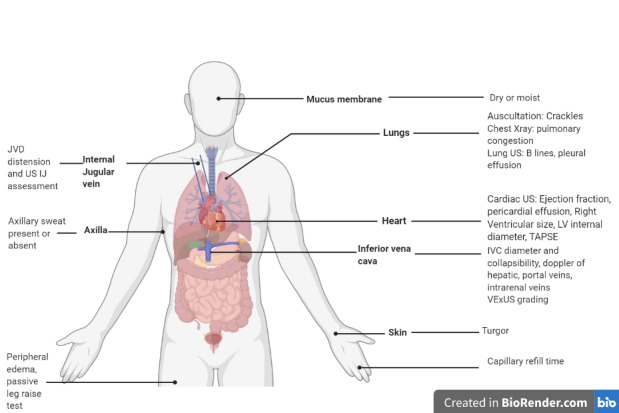
Schematic of Integrated Volume Assessment (Created with BioRender.com).

**Figure 6:**
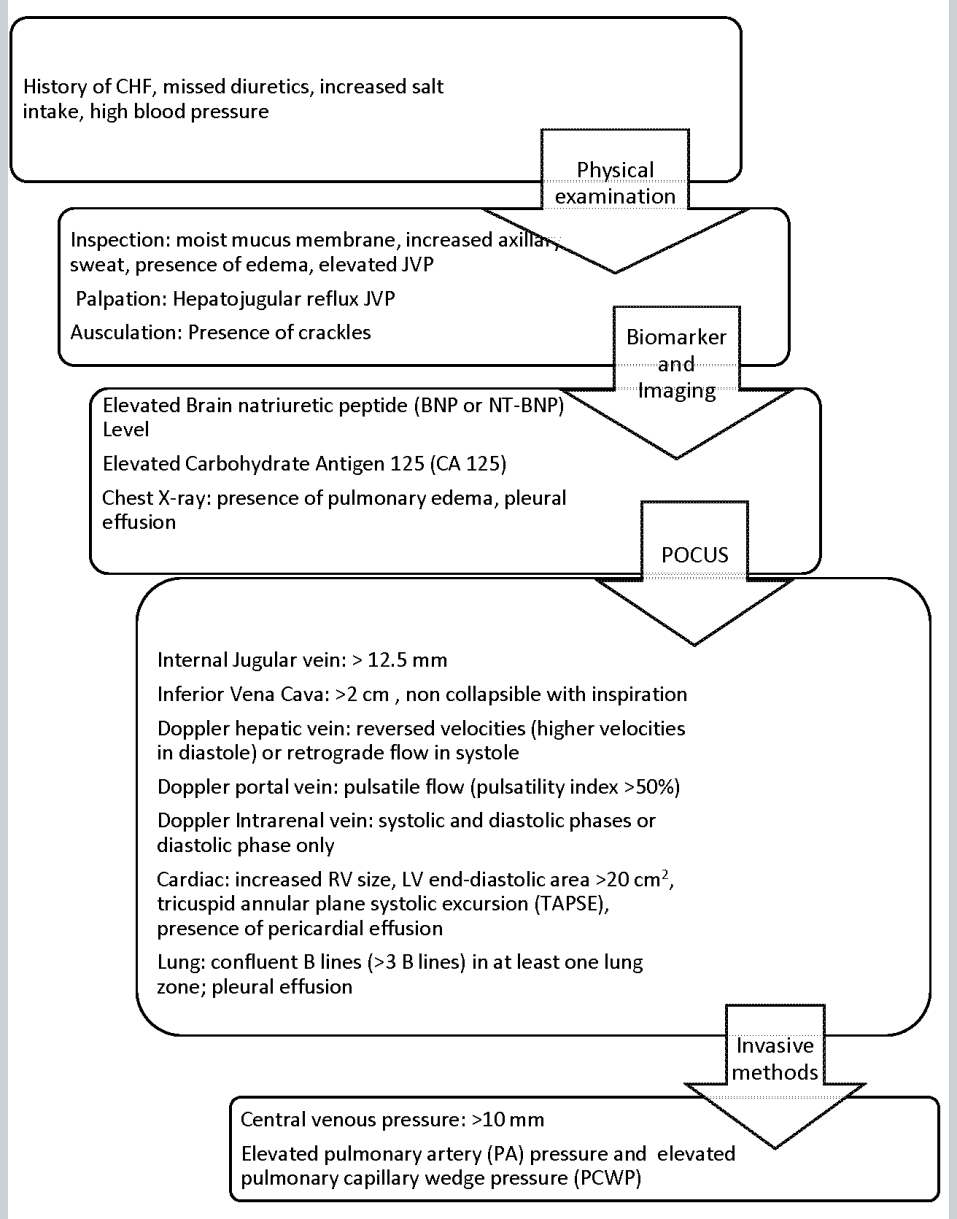
Algorithm for Systemic Approach to Volume Assessment: This is an algorithm that we propose will help with systematic approach to integrative volume assessment for diagnosing hypervolemia. Assigning a score system to each of the findings with higher scores to cardiac, lung and vascular ultrasound for volume assessment would be very useful tool to precisely diagnose volume status. Such tool needs to be studied and validated before widespread use. This approach will integrate POCUS to conventional methods and eliminate the shortcomings of individual methods.

We propose a stepwise approach to the volume assessment starting with conventional physical examination as feasible followed by combination of various POCUS methods (Figure 5 and 6). We will have a very good diagnostic tool if we use a scoring system with all the available physical examination and POCUS findings to decide on volume status using an aggregate score. We suggest assigning more weight to POCUS findings because of their ability to predict the volume status better than physical examination findings. Integration of these conventional and POCUS findings in a systematic way and using scoring system would provide valuable information in volume status management much closer to precision than use of these individually. Future clinical score development and validation using all these conventional and POCUS variables would be necessary and useful clinical practice tool. This is only a proposed scoring tool and needs to be validated. Future studies are recommended to validate such tool if it were to be developed. 

## Limitations

All these methods are subject to examiner or operator’s knowledge, skills and bias in addition to patient specific characteristics and hence caution should be used when interpreting these individually. If used by inexperienced operators, POCUS can provide inaccurate findings and lead to wrong diagnosis and error in management. Lack of proper training, appropriate credentialing process and ongoing quality assurance before widespread use can lead to failure to identify pitfalls in the techniques and cause patient harm. Currently we do not have external validation for POCUS findings nor validation with the clinical outcomes which can lead to incorrect diagnosis and prognostication. 

## Conclusion

POCUS has provided an important clinical tool to support our clinical examination and conventional methods in assessment of intravascular volume. None of the tools currently available provide perfect assessment without any pitfalls when used alone. However, with integration of all the pertinent clinical and POCUS tools using a scoring system, we can ensure precise volume assessment. With enforcement of validation methods, proper training, and formalized credentialing process, we can ensure its accuracy and reliability. In the meantime, recognizing the limitations, we should encourage wise integrated use of the various clinical and POCUS methods in our daily practice to provide precision care to our patients. 

## Disclosures

This manuscript is original and is not under consideration for publication elsewhere. The authors do not have any conflicts of interest or financial disclosure. 
